# Understanding the spatial–temporal variation of human footprint in Jiangsu Province, China, its anthropogenic and natural drivers and potential implications

**DOI:** 10.1038/s41598-020-70088-w

**Published:** 2020-08-07

**Authors:** Feixue Shen, Lin Yang, Xianglin He, Chenghu Zhou, Jonathan M. Adams

**Affiliations:** 1 School of Geography and Ocean Science, Nanjing University, Nanjing, 210023 China; 2 State Key Laboratory of Resources and Environmental Information System, Institute of Geographical Sciences and Natural Resources Research, CAS, Beijing, 100101 China

**Keywords:** Conservation biology, Social evolution, Environmental sciences

## Abstract

Understanding the spatial and temporal patterns of human pressures provides a foundation for understanding interactions between human and environment and managing human activities for a sustainable development. This study is the first attempt focused within China at calculating the spatial–temporal human footprint and its driving forces in a highly urbanized area with intensive human activities. Population, land use, night-time lights, and road impacts were used to generate human footprint maps of Jiangsu Province for 2000, 2010 and 2015 with a resolution of 1 km * 1 km. Five natural drivers and four anthropogenic drivers were employed to construct generalized additive models for explaining the spatial variation of human footprint and its change. It shows that a large difference is between the human footprint in northern and southern Jiangsu, and the pattern of human pressures conforms to the “Matthew effect”, with spatial aggregation of high human footprint areas accelerating. Slope, industrialization level are significant in explaining the spatial variation of human footprint in 2000, 2010 and 2015. The effect of natural drivers decreases for explaining the human footprint over time. Furthermore, annual precipitation, mean annual temperature and urban per capita disposable income are also significant drivers for human footprint in 2010 and 2015. And the increasing of human footprint slows with increasing of industrialization level. The difference of industrialization level and urban income between northern and southern Jiangsu mainly caused different driving pattern for human footprint and its change. Our study has generated new insights on the interaction pattern between human and nature in highly developed regions based on the human footprint concept, and can provide references for managing human activities in similar regions rapid socioeconomic development.

## Introduction

Humans are irreversibly changing the physical environment that supports them, for the sake of rapid development of human society^1–3^. Abundant evidence shows that human activities are becoming more intensive and diverse, exerting greater pressures on ecosystems both locally and globally^4–7^. Understanding the spatial and temporal patterns of human pressures^8^ on the Earth and its anthropogenic and natural drivers provides useful reference for better understanding interactions between human and environment and managing human activities for a sustainable development.

Earlier researchers mainly used population data to represent human pressure due to that it is a main underlying driving factor^9^^,^ and identify connections between changes of populations and environmental and social effects^10^. With the interactions between human and environment becoming more and more complex, land use has come to be considered as a useful way to represent different interactions that change natural environmental processes. Various researchers have assessed the impact of one or several types of land use on natural environment to express human pressures throughout region and globe^11–13^. However, using land use types is not able to adequately describe the complexity of human pressures due to the large heterogeneity of human activity types and intensities within one land use type^4^.

With recent advances in data acquisition techniques, human pressures have been mapped using more sophisticated techniques. The ‘human footprint’ is currently one of the most widely used concepts in mapping human pressures^14^. The concept of the human footprint was first proposed by Sanderson, et al. in 2002. It expresses human pressures as the sum of a continuum of human influence stretched across the land surface, revealing through its variation the major patterns of human influences on nature. Based on this concept, Sanderson, et al. (2002) generated the first global human footprint map, and the concept has since been applied in many other studies. For example, Haines et al.^15^ used human footprint data to assess landscape level conservation efforts. Burton et al.^16^ used such data for finding the relationship between human footprint and biodiversity and Woolmer et al.^17^ rescaled the human footprint for conservation planning at an eco-regional scale. Later, Venter et al.^18^ refined the above human footprint concept by distinguishing the effects of roads, railways and navigable waterways on accessibility and produced global human footprint maps comparing the years 1993 and 2009.

With application of the human footprint concept, many researchers produced human footprint maps at regional scales^17,19^. These studies mainly focused on ecosystems with largely natural landscapes, such as Tibet^20^^,^ and natural conversation districts^21^. By contrast, the human footprint for highly urbanized regions has been rarely studied. This seems a major omission, as over the past decades, many areas of the world have experienced varying extents of urbanization. Intense urbanization improves social development in many ways, but often causes severe environmental degradation and detracts from the benefits for overall human welfare. Assessing spatial–temporal variation of human footprint in rapidly urbanizing areas, and understanding its anthropogenic and natural drivers will help us find the key areas of conflicts between social development and nature environment protection, and thus provide guidance for managing human activities for a balance between natural environment with socio-economic development. In this paper, we take Jiangsu Province, China as a typical example of highly developed areas. Jiangsu Province exhibits large areas of productive agricultural lands, and also is one of the most developed provinces in China. The objectives of this paper are to (1) examine the spatial–temporal variation of human footprint in Jiangsu Province for a time period of 2000–2015, and (2) understand the anthropogenic and natural drivers of human footprint in Jiangsu Province.

## Results

### Spatial–temporal variation of human footprint in Jiangsu Province

**Figure 1:**
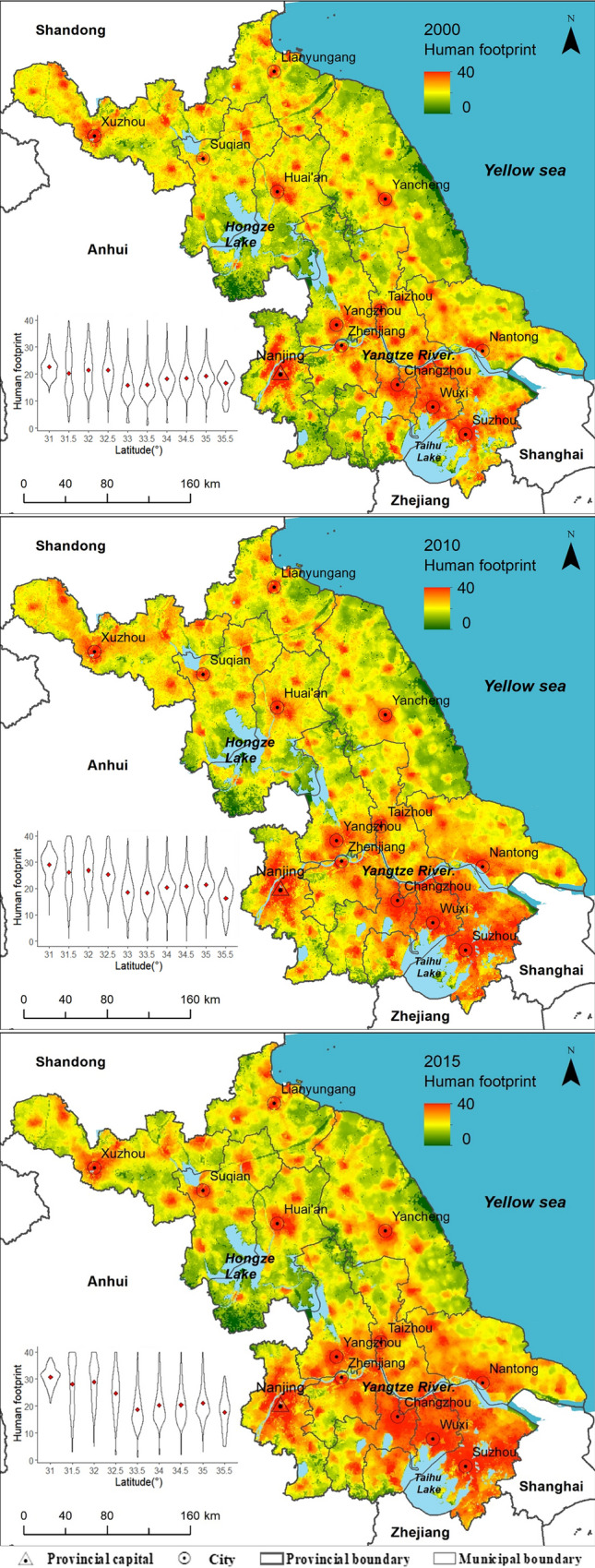
Human footprint of Jiangsu Province in 2000, 2010 and 2015. Spatial–temporal variation of human footprint and the violin plots^22^ of human footprint along every half latitude, the red point in each violin plot is the mean human footprint at each latitude interval. The human footprint maps were produced using ArcGIS 10.6 (https://desktop.arcgis.com/en/arcmap).

According to the available sources for calculating human footprint, the human footprint of Jiangsu in 2000, 2010, and 2015 were calculated with a resolution of 1 km * 1 km, shown in Fig. 1. We can see that there is a clear difference between northern and southern parts of Jiangsu Province, with boundary line in terms of human footprint between north and south lying at approximately 32.5°N. Generally, northern Jiangsu has lower human footprint values than southern Jiangsu, and the difference between north and south becomes more obvious over time.

The high human footprint index areas in northern Jiangsu are particularly centered around cities and scattered. The high human footprint index areas in southern Jiangsu are connected, especially in 2010 and 2015, except the western area where the capital of the province (Nanjing) is located. Furthermore, we can see that the human footprint in most coasts in northern Jiangsu (except Lianyungang, an important port city) and areas around Honze Lake (The national ecological function reserve of water resource of Eastern South-to-North Water Transfer Project) are not high. By contrast, the human footprint around the Taihu Lake, Yangtze River and even the protection areas of the estuary of the Yangtze River (The national ecological function reserve of Yangtze River estuary) are high and increase quickly from 2000 to 2015. This indicates that rapid social development is threatening the protection of the natural environment in southern Jiangsu.

**Figure 2:**
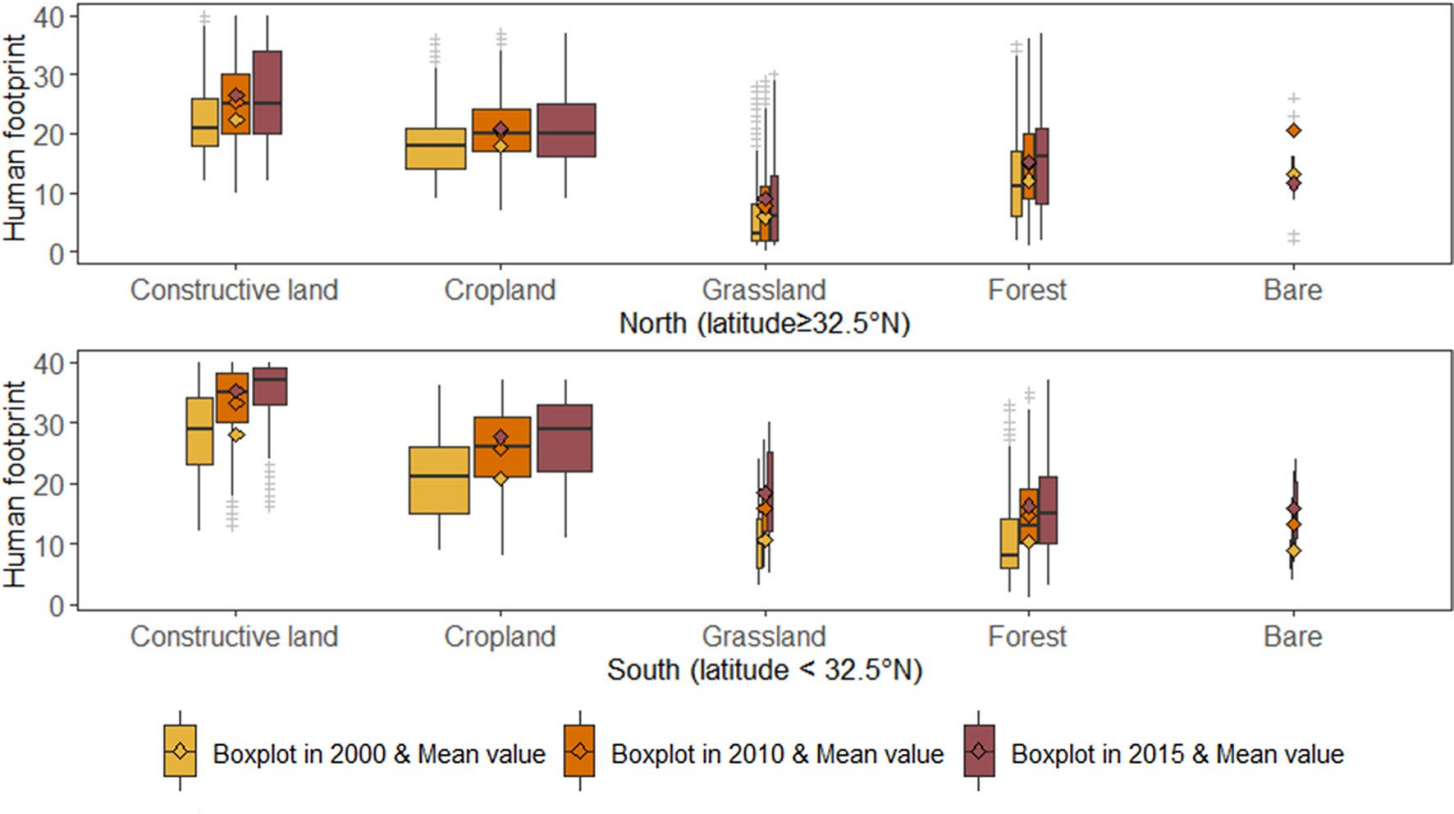
Boxplots of human footprint in different land use types for southern and northern Jiangsu in 2000, 2010 and 2015, the box width of each land use type is proportional to its area in each year.

Constructive land carries the largest human pressure in both northern and southern Jiangsu, followed by cropland which has the biggest area in either northern or southern Jiangsu (Fig. 2). The rapid development of human footprint in nearly all land use types and the expansion of constructed land in southern Jiangsu led to the spatial heterogeneity of human pressure between south and north. Although human footprint generally increased in every land use type in northern Jiangsu over time, the average human footprint value in most land use types keeps the same from 2010 to 2015. This indicates that the interference of human activities in northern Jiangsu slows down from 2010 to 2015 while human activities in southern Jiangsu are growingly intensive over time.

**Figure 3:**
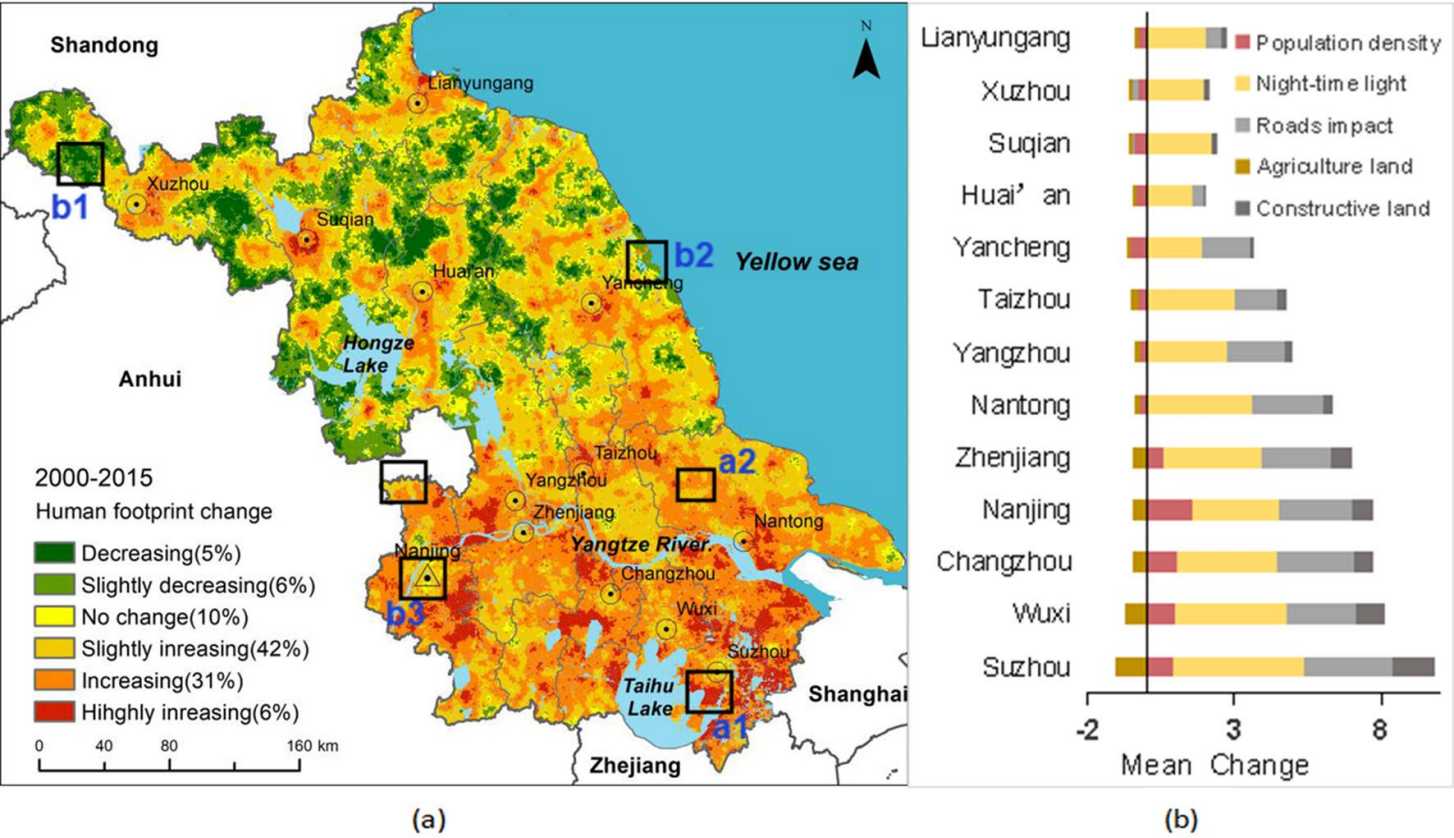
Change in human footprint from 2000 to 2015, (**a**) change of human footprint at a pixel scale. This map was produced using ArcGIS 10.6 (https://desktop.arcgis.com/en/arcmap). (**b**) the mean human footprint of each human pressure variable of the 13 municipal administrative districts of Jiangsu Province for 2000 and 2015.

**Figure 4:**
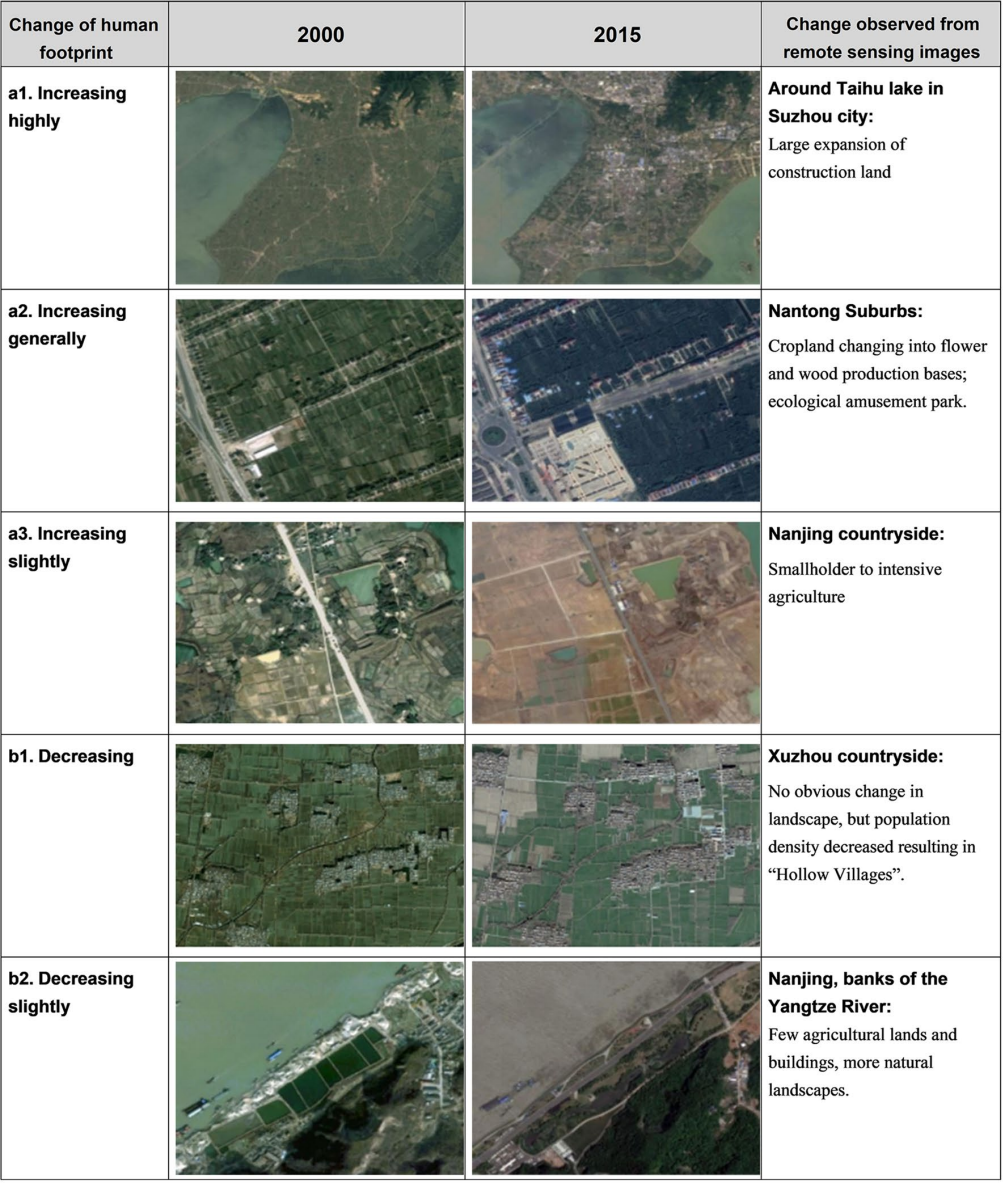
Images of 2000 and 2015 downloaded from Google earth representing changes of human footprint with different levels. Images (**a1**: 2000, 2015): Google, Landsat/Copernicus. Image (**a2**: 2015): Google, CNES/Airbus. Images (**a2**: 2000, **a3**: 2000, 2015, **b1**: 2000, 2015, **b2**: 2000, 2015): Google, Maxar Technologies.

Figure 3 shows the spatial distribution of human footprint change from 2000 to 2015. The change over time of the human footprint conforms to the “Matthew effect”, that is the higher the initial human footprint, the stronger the rate of increasing of the human footprint. This is clearly observed from the changing human footprint in southern Jiangsu. Furthermore, the aggregation effect of the human footprint in southern Jiangsu might play an important role in increase of the human footprint in this area. We downloaded several satellite images from Google representing different degrees of change in human footprint (Fig. 4). It can be seen from these test images that the human footprint maps are effective to represent human pressures and their change from 2000 to 2015 in Jiangsu.

Most areas in Jiangsu indicate an increase (79% of the total coverage of the province) of human pressure on the environment (Fig. 3a). The mean change over time in human footprint decreases from southern municipal districts to northern municipal districts of Jiangsu (Fig. 3b). The high increases of human footprint (37% of the total coverage of the province) are mainly due to the increase of night-time light, road impact and constructive land (Figs. 3b, 4a1–a3). The largest land use transforming from agricultural land to constructive land from 2000 to 2015 was observed based on land use data of Jiangsu. Agricultural area decreased more in southern Jiangsu than northern Jiangsu (Figs. 3b, 4a2), and increasing of population density occurred in most of the southern cities. This is consistent with the rapid urbanization process of Jiangsu and a higher degree of urbanization in south than in north^23^. Around 11% of the province mostly in the northern Jiangsu shows a decreased human footprint, mainly due to decreasing rural population (Figs. 3a, b, 4b1). This probably can be explained by the phenomenon that peasants drifted to cities to engage in secondary and tertiary industries for better incomes (Jiangsu Urbanization Development Report 2014). The decreasing of agricultural labor may cause the food production problem, or the government needs to change the agricultural management mode from family farming to large-scale cultivation. The decreasing or slightly increasing human footprint around the Hongze Lake and national Yancheng rare birds Nature Reserve in north is mainly due to the policy of designating national ecological function reserve and wildlife reserve^24^. Contrary to most of areas in south, small areas in capital of the province (Nanjing) undergoing a slightly decreasing in human footprint due to the policy to protect natural environment (such as revert cultivated land to wetland) (Fig. 4b2). It indicates that policy play a role in protecting natural environment from human pressures.

### Analysis on drivers of human footprint

#### Generalized additive models (GAM)

The explanatory statistics of the GAM models constructed using all drivers in different categories are shown in Table 1. For human footprint in 2000, the model constructed using all natural drivers performs better than the model constructed using all anthropogenic drivers in explaining spatial differences of human footprints with a deviance explained index (DE) of 80.6% versus 61.7%. For human footprint in 2010, anthropogenic drivers explain human footprint a little better than natural drivers with a DE of 89.4% versus 85.2%. For human footprint in 2015, anthropogenic drivers explain human footprint in 2015 more than natural drivers with a DE of 91.7% versus 79%. It indicates a more and more important role of anthropogenic drivers in explaining spatial differences of human footprint from 2000 to 2015. As for explaining the change of human footprint from 2000 to 2015, either natural or anthropogenic drivers obtained a high DE. When considering natural and anthropogenic drivers together, the explaining abilities of constructed models are all better than using only natural or anthropogenic drivers, and the constructed model of human footprint in 2015 and change of human footprint even generated DE higher than 97%. Therefore, natural or anthropogenic drivers are all necessary when explaining the spatial–temporal variation of human footprint.

**Table 1:** Results of the GAM models using different drivers without variable selection.

Human footprint	Models constructed using all natural drivers			Models constructed using all anthropogenic drivers			Models constructed using all natural and anthropogenic drivers		
	Deviance explained (%)	R-sq. (adj)	GCV	Deviance explained (%)	R-sq. (adj)	GCV	Deviance explained (%)	R-sq. (adj)	GCV
2000	80.6	0.71	6.01	61.7	0.56	6.93	91	0.83	4.69
2010	85.2	0.81	5.46	89.4	0.83	5.59	95	0.92	2.64
2015	79	0.73	11.3	91.7	0.89	4.64	97	0.95	2.89
Change from 2000 to 2015	86	0.83	1.65	92.8	0.91	0.93	97.3	0.95	0.73

**Figure 5:**
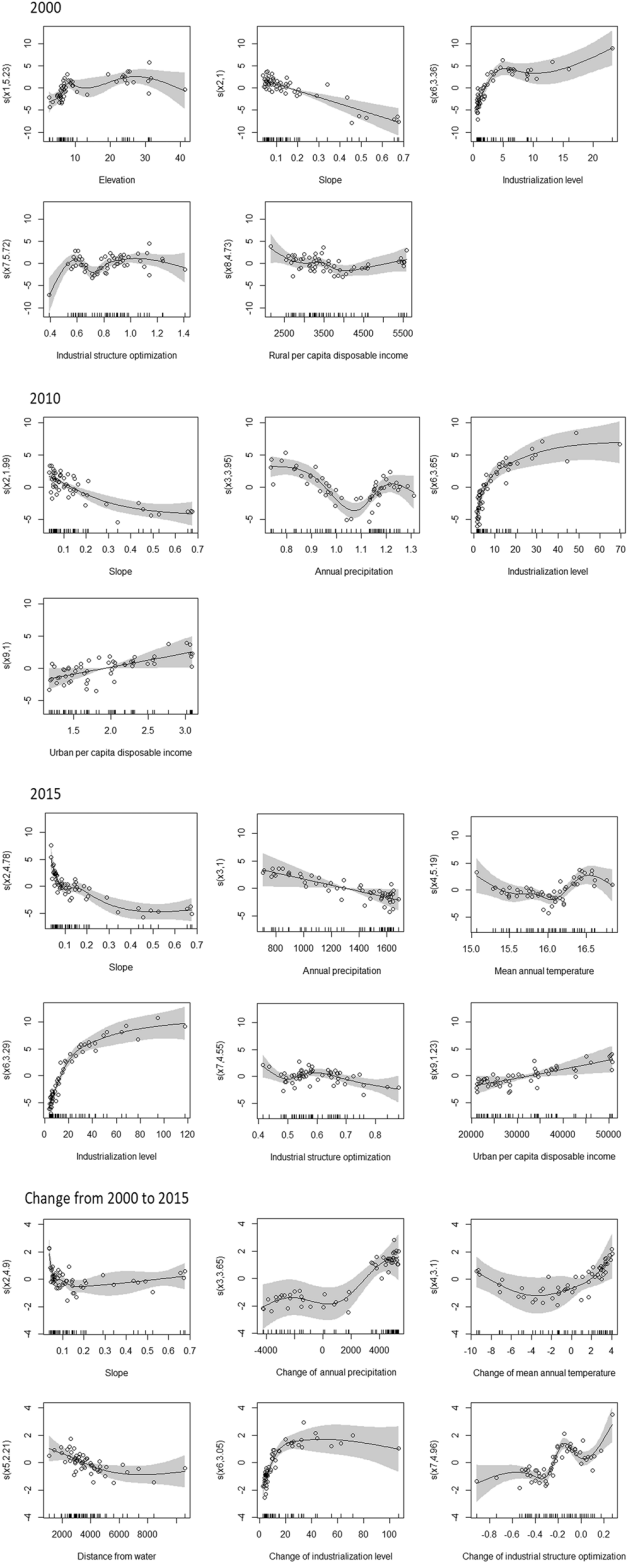
Smooth function estimates (est.) of the explanatory variables with the confidence bands using a shaded gray region and partial residuals using filled circles for the adjusted GAM of human footprint in 2000, 2010 and 2015 and human footprint change. The x-axis is the value of each driver. The y-axis, s (*x*, *n*), is the component smooth fitting value, in which *n* is the freedom of driver *x*.

After significance test of all the 9 variables, the adjusted models using the significant drivers are shown in Table 2. The deviance explained by the three adjusted GAMs are high, especially the model of human footprint in 2015 and the change of human footprint. The important natural and anthropogenic drivers for human footprint in each year and its change from 2000 to 2015 are a bit different. The industrialization level is the most important driver for human footprint in each year, followed by slope (Fig. 5). The two climate variables play an increasingly important role in explaining human footprint over time. This may indicate the interactive effects between human activities and climate. The urban per capita disposable income becomes a significant driver for human footprint in 2010 and 2015 while the rural per capita disposable income is significant for human footprint in 2000. As for the change of human footprint, slope, distance from water, change of annual precipitation, change of mean annual temperature, change of industrialization level, change of industrial structure optimization are the 6 significant drivers for explaining the change of human footprint from 2000 to 2015.

**Table 2:** Natural and anthropogenic drivers selected in the adjusted GAMs.

Adjusted models using significant natural and anthropogenic drivers				
	HF in 2000	HF in 2010	HF in 2015	Change of HF from 2000 to 2015
Elevation	***	/	/	/
**Natural drivers**				
Slope	***	***	***	*
Annual precipitation	/	***	*	**
Mean annual temperature	/	/	***	.
Distance from water	/			*
**Anthropogenic drivers**				
Industrialization level	***	***	***	***
Industrial structure optimization	***	/	*	***
Rural per capita disposable income	.	/	/	
Urban per capita disposable income	/	*	*	/
Deviance explained	88%	93%	97%	97.2%
R-sq. (adj)	0.81	0.92	0.95	0.95
GCV	4.23	2.34	2.59	0.63

Industrialization level = Added value of secondary industry/Added value of primary industry.

Industrial structure optimization = Added value of tertiary industry/Added value of secondary industry.

***p < 0.001; **p < 0.01; *p < 0.05; **.** : *p* < 0.1, /: Not significant.

The smooth function plots of each significant driver for the adjusted GAMs are shown in Fig. 5. All the human footprint in 2000, 2010 and 2015 decrease with increasing of slope, and the decreasing rate of slope for human footprint in 2000 keeps nearly the same while the decreasing rate of slope for human footprint in 2010 and 2015 generally becomes smaller and smaller. This shows that the effect of slope becomes smaller for explaining the human footprint at relatively higher slopes in 2010 and 2015 compared with in 2000, this may be explained by the effect of policy of “returning farmland to forests” and “relocating the poor”. In contrast, the human footprint in 2000, 2010 and 2015 increase with increasing of the industrialization level. It is also shown that industrialization level plays an increasingly important role from 2000 to 2015. It illustrates that higher industrialization of southern Jiangsu leading to more constructive land and attracting more workers caused a larger increase of human footprint in southern Jiangsu than in northern Jiangsu (see Supplementary Fig. S1 online). The increasing rate of human footprint with increasing of industrialization level slows down in 2015, which may indicate that the effect of industrialization level decreases when human footprint becomes large. At the same time, the human footprint in 2010 and 2015 increases with the increasing of urban per capita disposable income, which indicates that urban wealth causes more human pressure on environment in 2010 and 2015. The relationship between human footprint in 2000 and rural per capita disposable income is a bit complex. It may show that rural wealth only caused more human pressures when it up to some value in 2000. The fluctuate effect of the industrial structure optimization level for explaining human footprint in 2000 and 2015 may be because of the numerous types of tertiary industry resulting in different human impact on environment. For example, the human pressure caused by tourism is less than that caused by catering industry. It implies that development of environmentally friendly tertiary industries is suggested for developing areas such as northern Jiangsu. The climate drivers work for human footprint in 2010 and 2015. We found that the effect of climate drivers on human footprint is different between northern and southern counties (Fig. 5). Most of samples with annual precipitation less than 1050 mm in 2010, annual precipitation less than 1500 mm in 2015 and mean annual temperature lower than 16.2 °C in 2015 are northern counties (see Supplementary Fig. S1 online). Human footprints in northern counties decrease with increasing of annual precipitation and mean annual temperature. However, human footprints in counties in southern Jiangsu have a complicated relationship with annual precipitation in 2010 as well as mean annual temperature in 2015. It shows that the increasingly high human footprint in southern Jiangsu may result in complicated interactive relationships between climate and human activities.

As for the change of human footprint, there is nearly no effect of slope on change of human footprint when slope is larger than 0.1°. The change of human footprint decreases with increasing of distance from water, and the effect of distance from water decreases when distance from water is lager than 3500 m. The change of human footprint increases with increasing of the industrialization level, and the increasing rate slows down when industrialization level is larger than 20. The relationship between change of human footprint and industrial structure optimization is fluctuated, this may because different types of tertiary industries impact human pressures in a different way. Climate change and the human footprint change seems to have a complicated relationship. This might reveal a co-relationship between human footprint change and climate change^25–27^.

## Conclusions

This study is the first attempt focused within China at calculating the spatial–temporal human footprint and its driving forces in a highly urbanized area with intensive human activities. It reveals the development process of human activity driven by the natural and social-economic environment. The main conclusions include: (a) a large difference is between northern and southern parts of Jiangsu Province in human footprint. The spatial pattern of human footprint conforms to the “Matthew effect”, with spatial aggregation of high human footprint areas accelerating the increase. (b) The accumulation of deviance explained (ADE) of GAMs using the significant natural and anthropogenic drivers are 88%, 93%, 97% and 97.2% for human footprint in 2000, 2010, 2015 and the change of human footprint from 2000 to 2015, respectively. The results show that the relationships between human footprint and its change from 2000 to 2015 and their driving factors are complex. Both slope and industrialization level are significant in explaining the spatial variation of human footprint in the 3 years. However, the effect of natural drivers decreases for explaining the human footprint over time. Furthermore, annual precipitation and mean annual temperature play an increasingly important role in explaining human footprint in 2010 and 2015, and urban per capita disposable income are also significant drivers for human footprint in these 2 years. The increasing of human footprint slows with increasing of industrialization level. Higher level of industrialization doesn’t necessarily cause high human footprint when industrialization level reaches a high value. This is because of the diminishing marginal utility for one hand. For the other hand, people start to protect environment with socio-economic development and related policy promulgation. (c) The different combinations of natural and anthropogenic factors result in different spatial pattern of human footprint and its temporal change. The difference of industrialization level and urban income between northern and southern Jiangsu mainly caused different driving pattern for human footprint and its change. Higher industrialization of southern Jiangsu leading to more constructive land and attracting more workers caused a larger increase of human footprint in south than in northern Jiangsu.

Suggestions for the sustainable development of Jiangsu province can be provided based on the spatial characteristics of human footprint in Jiangsu. Northern Jiangsu is one important grain producing area in China and southern Jiangsu is one of the most developed areas in China. Due to the attraction of industrial development in southern Jiangsu, northern Jiangsu is facing the challenge of agricultural labor loss. Consequently, the ecological safety of southern Jiangsu may be severely threatened by further increased human disturbs on nature. Therefore, for northern Jiangsu, it is necessary to improve the agricultural production efficiency through recovering soil fertility, promoting agricultural mechanization, and changing the agricultural management mode from family farming to large-scale cultivation, etc. Meanwhile, policies can be properly designed to attract population to return to north. The tertiary industry (e.g., rural tourism industry or ecological education industry), which takes both environment and economic into account are recommended for northern Jiangsu by taking advantages of its tourism resources, such as wetlands. For southern Jiangsu, policies on controlling of urban sprawl and protecting environment should be continued to relieve the pressure of cities. Furthermore, there is an urgent need for publicizing the public self-governance for ecological environment protection and recovering, for example, communities with high educational and income level can be guided to establish an autonomous management system of settlements ecology.

Our study has generated new insights on the interaction pattern between human and nature in highly developed regions based on the human footprint concept. It provides a foundation for understanding the natural and anthropogenic causation for human pressures in areas with rapid socioeconomic development, and thus can provide references for managing human activities in similar regions.

## Data and methods

### Jiangsu Province, China

Jiangsu Province is located in eastern coastal China between latitudes 30° 45′ N and 35° 20′ N and longitudes 116° 18′ E and 121° 57′ E (Fig. 1), This region has an eastern Asian monsoon climate. The mean annual temperature of the province is approximately 13.6–16.1 °C and the annual precipitation is about 1,000 mm. The main terrain of the province is plains, which account for 85% of the study area. Hilly land accounts for 15% of the area, with elevation ranging from 0 to 625 m. Due to natural conditions favorable for agriculture, Jiangsu Province has become one of China’s most important grain producing areas.

Jiangsu Province had a population of 73.27 million in 2000 and 79.76 million in 2015 (Statistics Bureau of Jiangsu Province, https://tj.jiangsu.gov.cn/), with an annual growth rate of 0.53%. Since the China’s policy of reform and opening to the world starting in 1978, Jiangsu Province has experienced a variety of social-economic changes. Since 2000, the total GDP of Jiangsu has always ranked in 2nd place of all provinces in China. The urbanization rate of Jiangsu has also continuously ranked above the national average, reaching 60.6% in 2010 and 66.5% in 2015. The degree of agricultural modernization is also one of the highest amongst the provinces of China.

### Mapping Human footprint

We followed the concept of “human footprint”^14^ to map human pressure of Jiangsu Province for several years. Five variables measuring the direct and indirect human pressures on environment of Jiangsu Province were collected (Table 3). According to the data availability of these variables (especially the road data) (Table 3), human footprint in 2000, 2010 and 2015 were produced. The five variables were weighted to values of 0–10 (0 for lowest human pressure and 10 for highest human pressure) according to estimates of their relative levels of human pressure following Sanderson et al. (2002) and Oscar Venter et al. (2016). Most of the resolution for the original data is 1 km. We resampled the variables with resolution of not 1 km to be 1 km using ArcGIS 10.2. Finally, all the pressures were summed together to create the standardized human footprint index.

**Table 3:** Geographical datasets used to map human footprint in Jiangsu.

Pressure	Year	Data sets	Resolution	Sources
Population	2000, 2010, 2015	Gridded Population density of the World (GPW), v4 (2000, 2005, 2010, 2015, 2020)	960 m	CIESIN: Center for International Earth Science Information Network (https://sedac.ciesin.columbia.edu)
Constructive land	2000, 2010, 2015	National land use database for China (1990, 1995, 2000, 2005, 2010, 2015)	1 km	RESDC: Data Center for Resources and Environmental Sciences Chinese Academy of Sciences (https://www.resdc.cn)
Agricultural land			1 km	
Nighttime Lights	2000, 2010, 2013^a^	Global DMSP-OLS Nighttime Lights Time Series 1992–2013 (Version 4)	1 km	RESDC^b^ (https://www.resdc.cn) DMSP-OLS (https://www.ngdc.noaa.gov/eog/dmsp)
Road impacts	1995^c^, 2010, 2016^d^	National road data (1995, 2010, 2016)	–	RESDC, 1995; (https://www.resdc.cn) State Bureau of Surveying and Mapping, 2010 (https://www.ngcc.cn/ngcc/), Geographical Information Monitoring Cloud Platform of China, 2016 (https://www.dsac.cn/)

^a^Note that the data sets of night-time lights do not have night-time lights data at the year of 2015. We used the available data of 2013 to represent that of 2015.

^b^Note that we collected the data on platform of RESDC, it transformed the resolution of original DMSP-OLS Nighttime Lights datasets to 1 km.

^c^Note that the road data of 2000 is not available, we used the road data of 1995 to represent the road data of 2000 assuming that the changing of roads in Jiangsu from 1995 to 2000 is few.

^d^Note that the road data of 2015 is not available, we used the road data of 2016 to represent the road data of 2015.

#### Population density

The number of people in an area is frequently cited as a primary underlying cause of human pressure^28^. Human population density used in this study was the Gridded Population of the World, Version 4 (GPWv4) data sets^29^. This data was released every 5 years since 2000. For representing the impact of human activities, pixels with the original human population density value of 0 was assigned to a score of 0. The areas with other population density values were grouped into 10 bins, and then values in each bin were coded from 1–10 successively. To make the population density of the 3 years comparable, the data of 2000 was grouped into 10 bins using the quantile classification method in ArcGIS 10.2, and the thresholds of the ten bins were used as standards to convert the population density data of 2010 and 2015. The same approach for assigning scores is taken for other continues variable, such as night-lights time and road impact.

#### Land use/cover

Human beings transform land for settlements, growing food, and producing other economic goods^30,31^. Different land uses differ in the extent to which they modify ecosystem processes^32–34^. The land use data was obtained from the China’s land use database developed by Environmental Sciences Chinese Academy of Sciences (RESDC). This data was released for every 5 years since 1990, and is reported as the most accurate land use remote sensing monitoring data product in China^35^. The original data was at a resolution of 1 km with 6 primary classes and 26 secondary classes. According to Sanderson et al. (2002), all the areas mapped as urban, rural settlement and industrial transportation construction land in the original dataset were given a highest score of 10. All areas mapped as cropland, garden land, farming ponds and reservoirs were assigned a score of 7, areas of all other land use types were assigned to 0.

#### Night-time lights

DMSP/OLS (Defense Meteorological Satellite Program-Operational Linescan System) sensors work at night to detect the gleam visible-near infrared (VNIR) radiance on the earth surface, and it can collect the night lights with intensity degree from the urban lights and even small-scale residential areas, traffic, etc. This data is a good complement to capturing a lot of flowing and unobtrusive human activities^36^. We downloaded the DMSP-OLS data with the original resolution of 30 arc-seconds on the RESDC for the year of 2000, 2010 and 2013. The digital numbers (DN) of night-time lights were assigned with scores by the method for the population density data.

#### Roads

As one of humanity’s most prolific linear infrastructures, beyond simply reducing the extent of suitable habitat, roads can act as population sinks for many traffic-induced activities and shrink the distance of human from nature^37,38^. It can be known that the closer distance from a location to a road, the greater impact of human activities on the environment of this location, furthermore, the difference of impact with distance varies among the road types. For example, the impact of a railway for a location would be larger than that of a country road when the distance is the same. Therefore, we calculated the road impact with considering both distance and road type.

First, we calculated the shortest distance of each pixel to every type of road using Path Distance tools of ArcGIS 10.2. The maximum distance in terms of the impact of roads was set to 15-km according to the study of Sanderson. This means when roads all beyond 15 km away from one pixel, this pixel would be assigned a score of 0. To measure the total road impact pressure of one pixel, we calculated the weighted sum of all the minimum distance of each road type following the formula is:

$$RTD = \mathop \sum \limits_{i = 1}^{6} D_{i} *M_{i}$$

where RTD represent the total impact distance of roads for one pixel, the smaller the value, the greater the impact of roads. MI is the adjusted coefficient for representing the impact of different road type with distance (Expressway: 0.2, Railway: 0.37, National road: 0.53, Provincial road: 0.8, Country road: 0.87, Other road: 1), which was determined according to the study of   Li et al.^20^. Di is the shortest path distance of the pixel to each road type calculated using ArcGIS 10.2.

### Driving analyze

#### Driver factors

We selected five natural and four anthropogenic factors impacting the spatial and temporal human footprint index in Jiangsu Province. The five natural variables are elevation, slope, mean annual temperature, annual precipitation and distance from water. Four anthropogenic drivers, industrialization level, industrial structure optimization^39,40^, rural per capita disposable income and urban per capita disposable income were selected for representing the social development level. The elevation and slope are extracted from the digital elevation model which is downloaded from Geospatial Data Cloud site, Computer Network Information Center, Chinese Academy of Sciences (https://www.gscloud.cn). Mean annual temperature and annual precipitation for 2000 and 2015 were downloaded from the Data Center for Resources and Environmental Sciences Chinese Academy of Sciences (RESDC) (https://www.resdc.cn), the distance from water of a pixel was calculated by the Path Distance tool in ArcGIS 10.2. We collected the rural and urban per capita disposable income and value- added of primary, secondary and tertiary industries of the 55 counties of Jiangsu Province in 2000, 2010 and 2015 from the Statistical Yearbook of Jiangsu Province. The industrialization level is calculated as the value-added of secondary industry divided by the value-added of primary industry. The industrial structure optimization is calculated as the value-added of tertiary industry divided by the value-added of secondary industry. These anthropogenic indices are independent of the human footprint calculated above for that these human pressure data were absolutely not used these four statistical data.

#### Generalized additive model (GAM)

GAM^41^ is an extension of generalized linear model (GLM) in which the linear predictor is given by a sum of smooth functions of independent variables^42,43^. The linear predictor of a GAM has a structure as follows,

$${\text{g}}\left( {mu_{i} } \right) = \theta + \mathop \sum \limits_{i = 1}^{n} f_{{i\left( {x_{i} } \right)}}$$

where the response variables *y*_*i*_ has an expectation of *mu*_*i*_ and *g* is a known monotonic ‘link’ function, *θ* is a constant, *f*_*i*_ is a smoothing function that describes the relationship between g(*mu*_*i*_) and the independent variables *x*_*i*_, and n is the number of variables.

GAM is flexible relative to strictly parametric linear or non-linear models for discerning effects of multiple factors^44^. GAM has been widely applied in detecting drivers and predicting spatial distributions of geographic elements/phenomena^45,46^. We thus chose GAM to detect and compare the driving effects of natural and anthropogenic driving factors for human footprint and its change in Jiangsu Province.

Three pools of drivers were developed, including natural drivers, anthropogenic drivers, natural and anthropogenic drivers. We first constructed GAM models for human footprint in 2000, 2015 and change of human footprint using all variables in each pool to compare the explanation effects of different drivers. The construction of a GAM model was mainly guided by generalized cross-validation (GCV), i.e. lower GCV values indicate better-fitted models. Besides, adjustment R square (R-sq. (adj)) was generated to evaluated the fitted models. The deviance explained (DE) was generated to examine the explanation ability of the sum effects of driving variables, where a higher DE value represents a better explanatory ability. Then, we constructed adjusted GAM models only using those significant variables within each pool with a backward stepwise method. We employed the p-scores to evaluate the significance of each driver and selected the significant drivers with a *p* < 0.1. We also plotted smooth functions of each significant drivers to examine their effect for explaining human footprint in each year and the change of human footprint from 2000 to 2015. The vertical axis in the plots is a relative scale indicating the effect of that explanatory variable on the dependent variable.

The mean value of every natural drivers and human footprint in the 55 counties of Jiangsu Province were calculated as the covariates *x*_*i*_ and response variables *y*_*i,*_ respectively. We constructed GAMs with the *mgcv* package in the *R* software. The univariate penalized cubic regression spline smooth function and an identity link function were used for *fi* and *g* in this study.

## Supplementary information

Supplementary file1.

## Data Availability

The datasets generated during and/or analyzed during the current study are available from the corresponding author on reasonable request.

## References

[1] Vitousek PM, D'Antonio CM, Loope LL, Rejmanek M, Westbrooks RG (1996). Introduced species: a significant component of human-caused global change. N. Z. J. Ecol..

[2] Halpern BS (2008). A global map of human impact on marine ecosystems. Science.

[3] Folke C (2011). Reconnecting to the biosphere. Ambio. J. Hum. Environ..

[4] Rounsevell MDA (2012). Challenges for land system science. Land Use Pol..

[5] Motesharrei S (2016). Modeling sustainability: population, inequality, consumption, and bidirectional coupling of the earth and human systems. Nat. Sci. Rev..

[6] Waters CN (2016). The Anthropocene is functionally and stratigraphically distinct from the Holocene. Science.

[7] Díaz S (2018). Assessing nature’s contributions to people. Science.

[8] Venter O (2016). Sixteen years of change in the global terrestrial human footprint and implications for biodiversity conservation. Nat. Commun..

[9] Phillipson DW, Butzer KW (1983). Archaeology as Human Ecology: Theory and Method for a Contextual Approach.

[10] Macdonald D (1998). Rediscovering geography: new relevance for science and society by Wilbanks. Geography.

[11] Zhuang D, Liu J (1997). Study on the model of regional differentiation land use degree in China. J. Nat. Resour..

[12] Di X, Hou X, Wang Y, Wu L (2015). Spatial–temporal characteristics of land use intensity of coastal zone in China during 2000–2010. Chin. Geogr. Sci..

[13] Chi Y, Shi H, Zheng W, Su J, Fu Z (2018). Spatiotemporal characteristics and ecological effects of the human interference index of the Yellow River Delta in the last 30 years. Ecol. Indic..

[14] Sanderson EW (2002). The human footprint and the last of the wild. Bioscience.

[15] Haines AM, Leu M, Svancara LK, Scott JM, Reese KP (2008). A theoretical approach to using human footprint data to assess landscape level conservation efforts. Conserv. Lett..

[16] Burton AC (2014). A framework for adaptive monitoring of the cumulative effects of human footprint on biodiversity. Environ. Monit. Assess..

[17] Woolmer G (2008). Rescaling the human footprint: a tool for conservation planning at an ecoregional scale. Landsc. Urban Plan..

[18] Venter O (2016). Global terrestrial human footprint maps for 1993 and 2009. Sci. Data.

[19] Ayram CAC, Mendoza ME, Etter A, Pérez-Salicrup D (2017). Anthropogenic impact on habitat connectivity: a multidimensional human footprint index evaluated in a highly biodiverse landscape of Mexico. Ecol. Indic..

[20] Li S (2018). Human footprint in Tibet: assessing the spatial layout and effectiveness of nature reserves. Sci. Total Environ..

[21] Tapia-Armijos MF, Homeier J, Munt DD (2017). Spatio-temporal analysis of the human footprint in South Ecuador: influence of human pressure on ecosystems and effectiveness of protected areas. Appl. Geogr..

[22] Hintze JL, Nelson RD (1998). Violin plots: a box plot-density trace synergism. Am. Stat..

[23] Ma, X. & Shen, Z. Study on the spatial pattern and evolution of urbanization, Jiangsu Province. *Econ. Geogr.* **5**, 783–786, 795 (2007)

[24] China.org.cn. https://www.china.com.cn/chinese/huanjing/280252.htm (2003)

[25] Huang C (2015). Changes in land use, climate and the environment during a period of rapid economic development in Jiangsu Province, China. Sci. Total Environ..

[26] Xia L (2015). Climate change characteristics in Jiangsu Province, 1960–2012. J. Glaciol Geocryol..

[27] Zhou F, Sun Z (2014). Variation of 1961–2010 summer temperature abnormal in Jiangsu and its circulation background features. J. Meteorol. Sci..

[28] Cincotta RP, Engelman R (2000). Nature’s Place: human population density and the future of biological diversity. Pac. Conserv. Biol..

[29] Center for International Earth Science Information Network—CIESIN—Columbia University. Documentation for the Gridded Population of the World, Version 4 (GPWv4). Palisades NY: NASA Socioeconomic Data and Applications Center (SEDAC). 10.7927/H4D50JX4. Accessed 10 Jan 2019 (2016).

[30] Foley JA (2005). Global consequences of land use. Science.

[31] Geist HJ, Lambin EF (2002). Proximate Causes and Underlying Driving Forces of Tropical DeforestationTropical forests are disappearing as the result of many pressures, both local and regional, acting in various combinations in different geographical locations. Bioscience.

[32] Lawler JJ (2014). Projected land-use change impacts on ecosystem services in the United States. Proc. Natl. Acad. Sci. U. S. A..

[33] Bauer C, Dubreuil A, Gaillard G (2007). Key elements in a framework for land use impact assessment within LCA. Int. J. Life Cycle Assess..

[34] Goudie AS (2000). The human impact on the natural environment. Trans. Inst. Br. Geogr..

[35] Liu J (2018). Spatio–temporal patterns and characteristics of land-use change in China during 2010–2015. Acta Geogr. Sin..

[36] Elvidge CD (1997). Mapping city lights with nighttime data from the DMSP operational linescan system. Photogramm. Eng. Remote Sens..

[37] Trombulak SC, Frissell CA (2000). Review of ecological effects of roads on terrestrial and aquatic communities. Conserv. Biol..

[38] Assisa JC, Giacominic HC, Ribeirob MC (2019). Road permeability index: evaluating the heterogeneous permeability of roads for wildlife crossing. Ecol. Indic..

[39] Haraguchi N, Martorano B, Sanfilippoc M (2019). What factors drive successful industrialization? Evidence and implications for developing countries. Struct. Change Econ. Dyn..

[40] Dong F (2019). The process of peak CO_2_ emissions in developed economies: a perspective of industrialization and urbanization. Resour. Conserv. Recycl..

[41] Hastie T, Tibshirani R (1986). Generalized additive models. Stat. Sci..

[42] Wood SN (2011). Fast stable restricted maximum likelihood and marginal likelihood estimation of semiparametric generalized linear models. J. R. Stat. Soc. B.

[43] Wood SN (2003). Thin plate regression splines. J. R. Stat. Soc. B.

[44] Wood SN, Fasiolo M (2017). A generalized Fellner-Schall method for smoothing parameter optimization with application to Tweedie location, scale and shape models. Biometrics.

[45] Marques DDS (2019). Selection of biochemical and physiological parameters in the croaker, micropogonias furnieri, as biomarkers of chemical contamination in estuaries using a generalized additive model (gam). Sci. Total Environ..

[46] Feng Y (2018). Evaluating land ecological security and examining its relationships with driving factors using GIS and generalized additive model. Sci. Total Environ..

