# Supplementary material for: Understanding the spatial–temporal variation of human footprint in Jiangsu Province, China, its anthropogenic and natural drivers and potential implications
Source: Sci Rep. 2020 Aug 7;10:13316. doi: 10.1038/s41598-020-70088-w (PMC7414874; doi:10.1038/s41598-020-70088-w)
Supplement: Supplementary file 1 — Supplementary file1. [file 41598_2020_70088_MOESM1_ESM.pdf]

Understanding the spatial-temporal variation of human footprint in Jiangsu Province, China, its anthropogenic and natural drivers and potential implications

Feixue Shen [1], Lin Yang[1,*], Xianglin He[1], Chenghu Zhou [1, 2], Jonathan M. Adams [1]

[1] School of Geography and Ocean Science, Nanjing University, Nanjing, 210023 China
[2] State Key Laboratory of Resources and Environmental Information System, Institute of Geographical Sciences and Natural Resources Research, CAS, Beijing 100101, China
[*]Corresponding author. yanglin@nju.edu.cn

2000

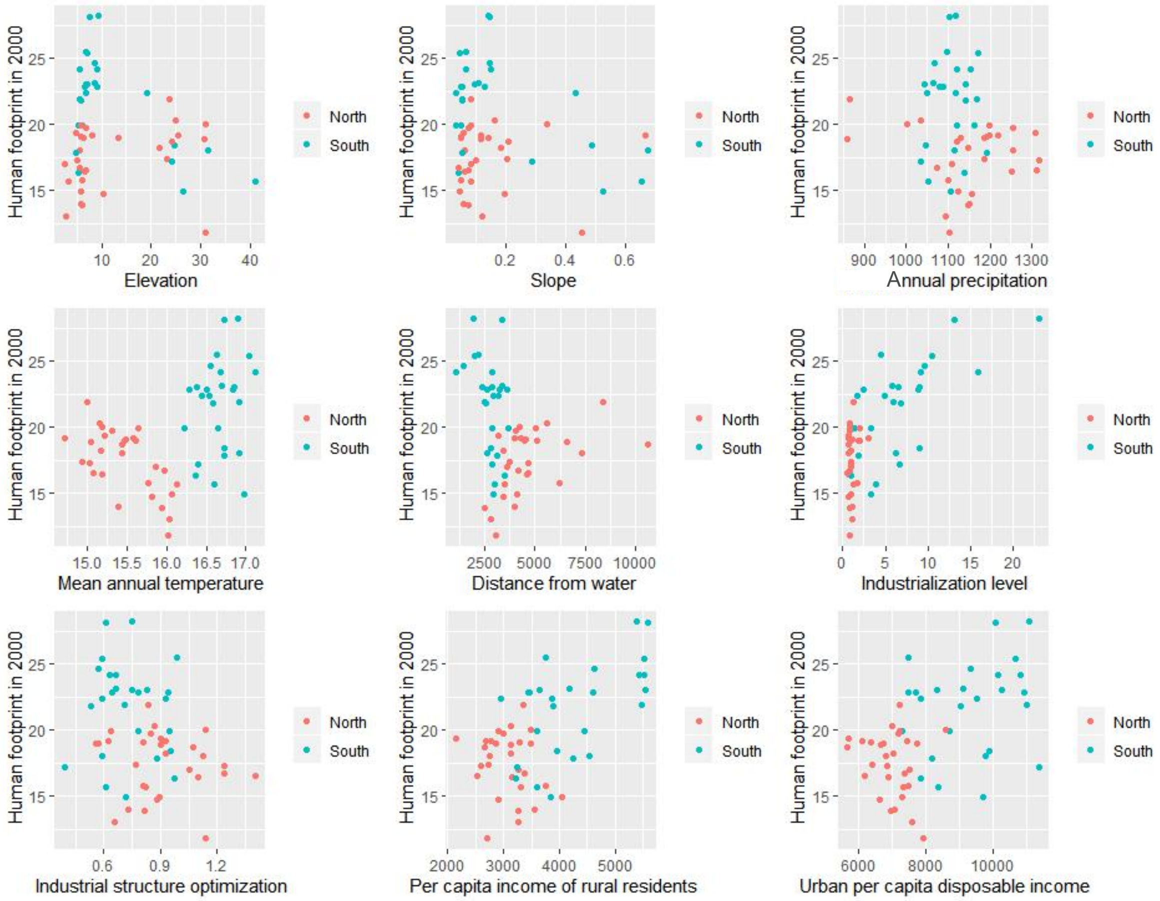

2010

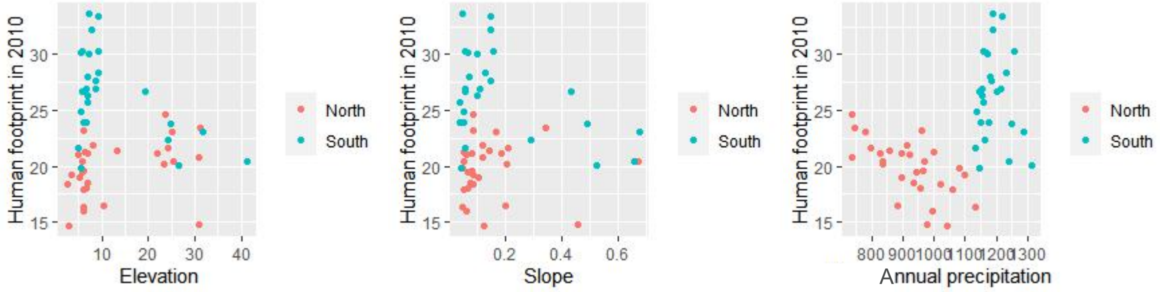

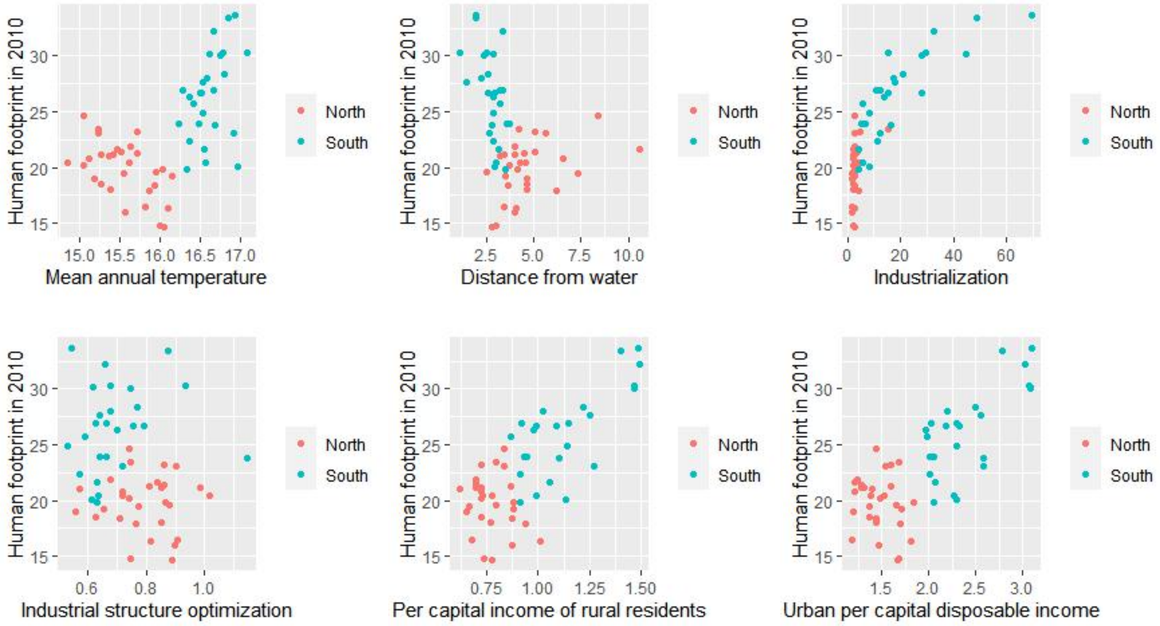
Human footprint in 2010
Mean annual temperature
North
South
Human footprint in 2010
Distance from water
North
South
Human footprint in 2010
Industrialization
North
South
Human footprint in 2010
Industrial structure optimization
North
South
Human footprint in 2010
Per capital income of rural residents
North
South
Human footprint in 2010
Urban per capital disposable income
North
South

2015

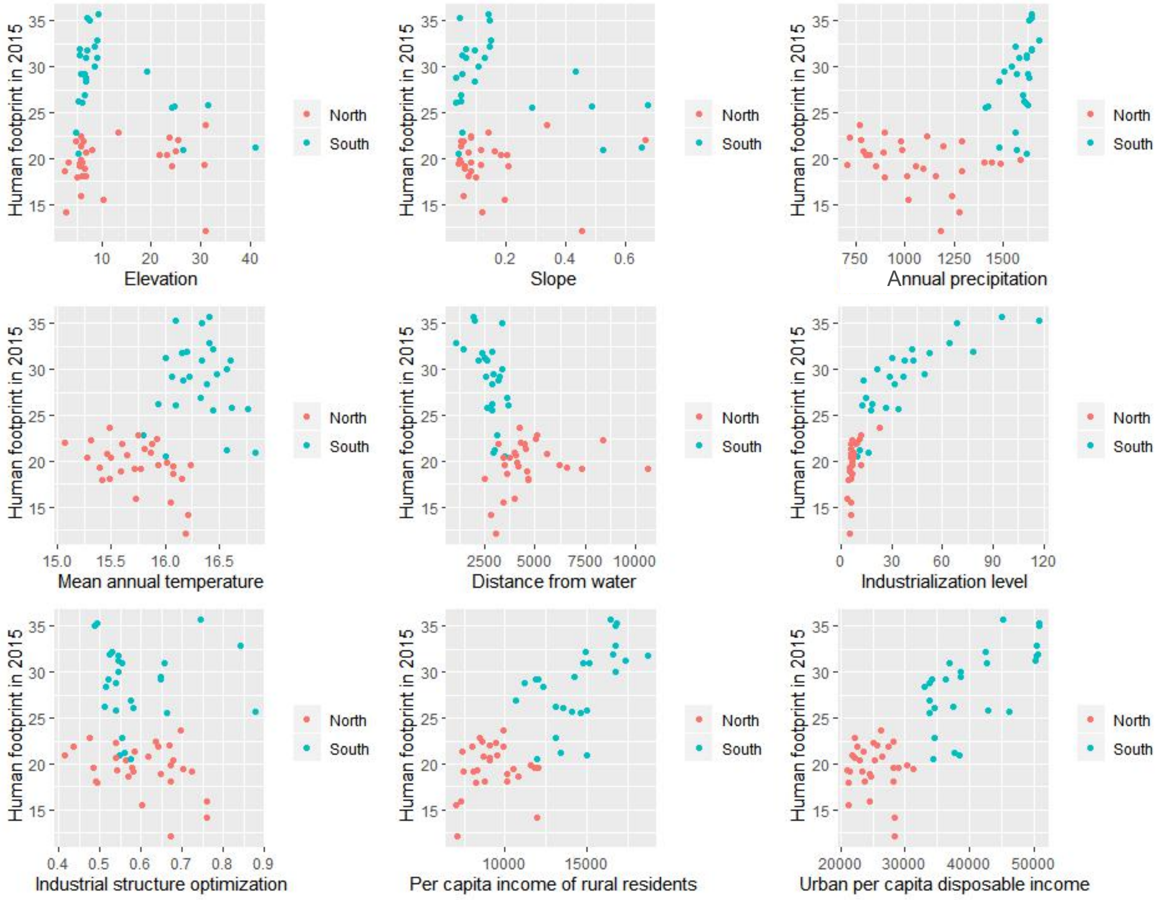
Human footprint in 2015
Elevation
North
South
Human footprint in 2015
Slope
North
South
Human footprint in 2015
Annual precipitation
North
South
Human footprint in 2015
Mean annual temperature
North
South
Human footprint in 2015
Distance from water
North
South
Human footprint in 2015
Industrialization level
North
South
Human footprint in 2015
Industrial structure optimization
North
South
Human footprint in 2015
Per capita income of rural residents
North
South
Human footprint in 2015
Urban per capita disposable income
North
South

Change from 2000 to 2015

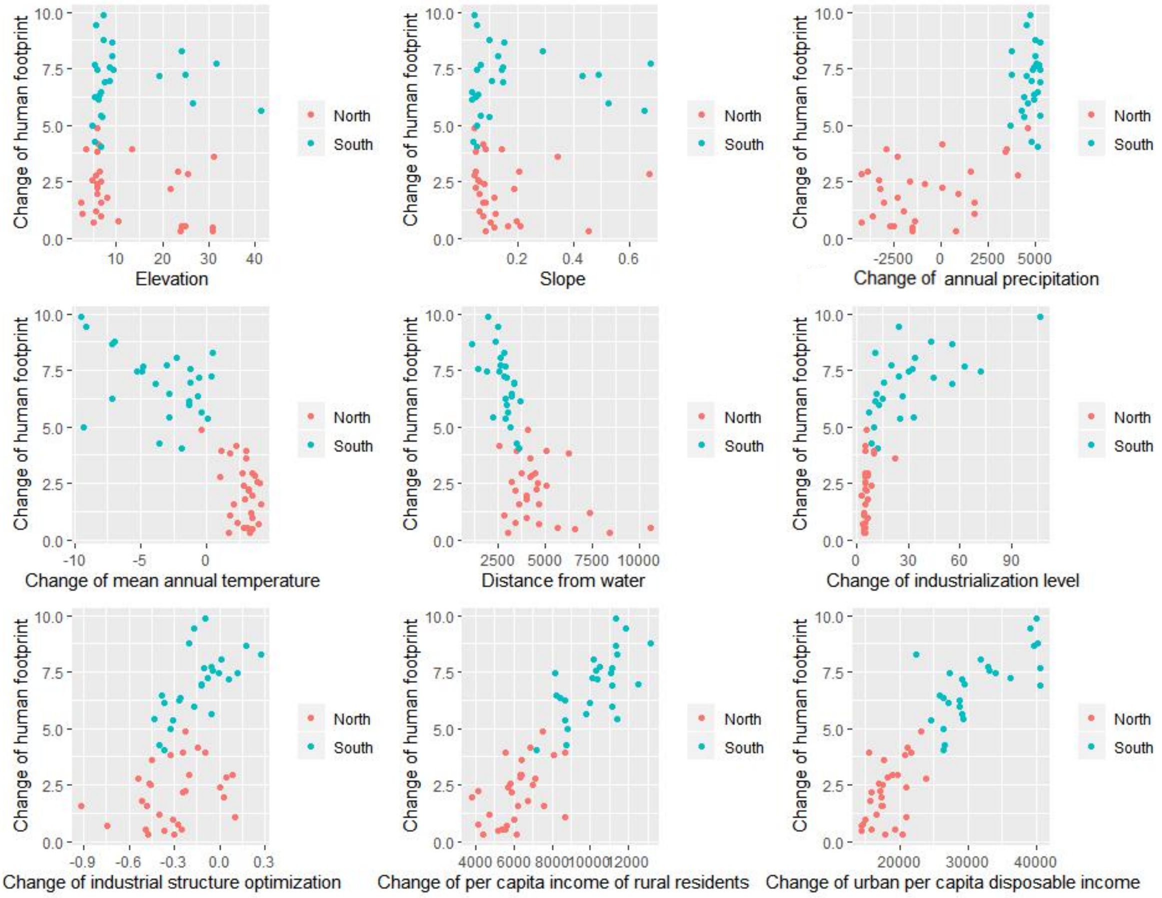

Supplementary Figure S1. Scatter plots of human footprint in 2000, 2010 and 2015 and human footprint change and the five natural and four anthropogenic drivers for the 29 north counties and 26 south counties.
